# Newly identified intronic and known pathogenic point mutations in *SLC34A3/NPT2c* cause hereditary hypophosphatemic rickets with hypercalciuria

**DOI:** 10.1016/j.gendis.2024.101318

**Published:** 2024-05-07

**Authors:** Yujing Sun, Yuan Liu, Xiaoli Zhang, Ling Jiang

**Affiliations:** Department of Endocrinology, Qilu Hospital, Cheeloo College of Medicine, Institute of Endocrine and Metabolic Diseases of Shandong University, Jinan Clinical Research Center for Endocrine and Metabolic Diseases, Jinan, Shandong 250012, China

Hereditary hypophosphatemic rickets with hypercalciuria (HHRH) is a rare autosomal recessive disorder characterized by hypophosphatemia, hypercalciuria, and recurrent nephrolithiasis, often resulting in rickets and osteomalacia.^1^ Mutations in the *SLC34A3*, which encodes the renal sodium-phosphate cotransporter NaPi-IIc, are the leading cause of HHRH. A case study of a 30-year-old female patient revealed she had compound heterozygous mutations in *SLC34A3*, including a novel intronic mutation, impacting gene splicing and reducing protein production. This underscores the critical role of *SLC34A3* in HHRH and broadens the understanding of its genotypic and phenotypic diversity. Genetic diagnosis is crucial in identifying this rare disease, as active vitamin D is contraindicated in HHRH patients due to the associated hypercalciuria and nephrolithiasis.

Our study highlights a unique HHRH patient with a novel intronic *SLC34A3* mutation. The patient, a 30-year-old female, initially sought medical attention at age 2 for leg bowing, bone pain, and recurrent kidney stones. Misdiagnosed with X-linked hypophosphatemia, she received phosphate supplementation and calcitriol treatment, maintaining normal serum phosphate levels but experiencing recurring kidney stones. She had orthopedic surgeries at age 12 and reached her final height of 142 cm by age 14 (−3.36 standard deviation). Genetic testing before conception did not identify any mutations. After pregnancy, she managed her phosphate levels with 0.5 μg calcitriol per day, discontinuing phosphate supplementation. During the physical examination, pronounced deformities in her lower limbs were evident (Fig. 1A). Laboratory data revealed normal serum phosphate of 0.76 mmol/L and an elevated alkaline phosphatase of 143 U/L. In terms of urinary measurements, her 24-h urine calcium was 10.52 mmol/24 h, and her urine phosphate was 28.11 mmol/24 h. Her family members' laboratory test results were normal (Table S1).

**Figure 1:**
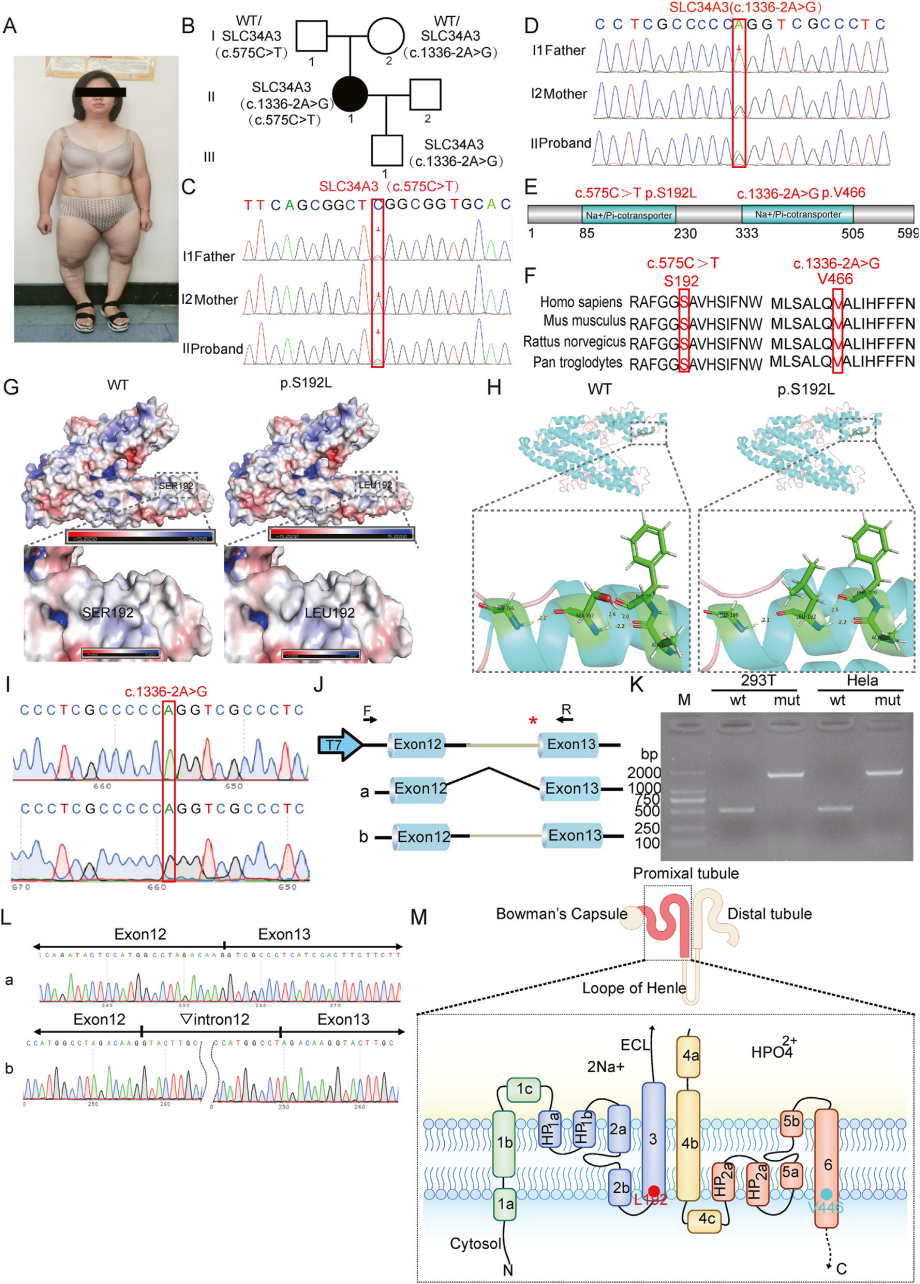
Comprehensive analysis of newly identified intronic and known pathogenic point mutations in *SLC34A3/NPT2c* causing hereditary hypophosphatemic rickets with hypercalciuria. **(A)** Clinical presentation. Remarkable deformities were evident during the physical examination. **(B)***Pedigree of the proband's family. The black circle denotes the proband.***(C)***Chromatogram of the proband (II1) showing the heterozygous****SLC34A3****c.575C > T variant (NM_080877) as present in the proband's father (I1).****(*D)***Chromatogram of the proband (II1) showing the heterozygous (NM_080877) c*.1336-2A > G *variant (NM_080877) as present in the proband's mother (I2) and son (III1).***(E)** Structural domains of SLC34A3. The c.575C > T (p.Ser192Leu) and c.1336-2A > G (splicing) mutations within SLC34A3 are localized in the Na+/Pi-cotransporter region, as predicted by InterPro and UniProt. **(F)** Evolutionary conservation of SLC34A3/NaPi2c. An analysis of evolutionary conservation at the amino acid positions corresponding to the c.575C > T (p.Ser192Leu) and c.1336-2A > G (splicing) mutations reveals high conservation, with these positions consistently occupied by serine across five different species. **(G)** Impact of SLC34A3 c.575C > T (p.Ser192Leu) mutations on protein surface potential. Electrostatic effects play a crucial role in protein structure and function. In the representation, blue denotes positively charged regions, red indicates negatively charged regions, and white signifies uncharged regions. This charge distribution is pivotal as it influences the binding interactions between molecules, with the strength of binding correlating with the magnitude of charge. **(H)** Influence of mutation on the tertiary structure of SLC34A3 c.575C > T (p.Ser192Leu). The mutation c.575C > T (p.Ser192Leu) is shown to have structural implications on the tertiary conformation of SLC34A3, potentially altering its overall three-dimensional structure. **(I**–**L)** Minigene assay using pcDNA3.1 vector in 293T and Hela cells. (I) Sequencing results of *pcDNA3.1-SLC34A3-WT* and *pcDNA3.1-SLC34A3(1336-2A > G)*. (J) Agarose gel electrophoresis of the PCR products in 293T and Hela cells (band a). (K) Schematic representation of the construction of *pcDNA3.1-SLC34A3-WT* and *pcDNA3.1-SLC34A3(1336-2>G)* splicing modes. Both *pcDNA3.1-SLC34A3-WT* and *pcDNA3.1-SLC34A3(1336-2A > G)* include exon 12, intron/exon boundaries, and exon 13. Exon 12 and exon 13 are regions with strong splicing recognition used for the splicing study. (L) Sequencing results of the PCR products using designed primers. **(M)** Localization and predicted topology of the SLC34A3/NaPi2c mutations c.575C > T p.S192L and c.1336-2A > G (splicing) p.V446.

We used targeted whole-exome sequencing to focus on OMIM (Online Mendelian Inheritance in Man)-listed genes related to nephrocalcinosis and nephrolithiasis. We identified two key mutations responsible for the patient's condition: a heterozygous c.575C > T (p.Ser192Leu) mutation and a c.1336-2A > G (splicing) mutation in *SLC34A3* gene. Segregation analysis confirmed that the proband's father (I1) had the c.575C > T variant, while the proband's mother (I2) and the proband's son (III1) both carried the c.1336-2A > G mutation (Fig. 1B–D). Our genetic assessment pinpointed *SLC34A3* mutations as the precise genetic diagnosis for nephrocalcinosis and nephrolithiasis, focusing on known OMIM-associated causes. We then assessed the frequency of SLC34A3 p.Ser192Leu using ChinaMap, which showed it to be approximately 0.0047%. To understand the functional impact of this mutation, we predicted its effect on the protein structure. The conserved domain of human SLC34A3 includes the Na^+^/Pi-cotransporter region, which actively transports phosphate into cells through Na^+^ cotransport in the renal brush border membrane (Fig. 1E). A conservative analysis confirmed high conservation of the amino acids at the c.575C > T p.S192L and c.1336-2A > G variant sites as serine (Ser) and valine (Val) across four scrutinized species (Fig. 1F).

Subsequent APBS (Adaptive Poisson-Boltzmann Solver) mapping analysis within PyMOL revealed that the surface charge of the Ser192 protein remained neutral after the mutation to Leu192, indicating minimal impact on the surface charge of the SLC34A3 protein (Fig. 1G). I-TASSER (Iterative Threading ASSEmbly Refinement) modeling and PyMOL mapping analysis showed changes in hydrogen bond distances between Ser192Leu and Phe189, with no significant alterations in distances between Ser192Leu and Ala88 or Ser192Leu and Gly196, implying a potential impact on the main chain structure of SLC34A3. Additionally, the mutation led to the disappearance of the original side chain, suggesting an influence on the side chain structure (Fig. 1H). In summary, these mutations are likely to affect the tertiary structure of the SLC34A3 protein.

To confirm the impact of the c.1336-2A > G variant on splicing, we conducted a minigene splicing experiment using pcDNA3.1 and pcMINI-C vectors. Reverse transcription PCR consistently showed that in both 293T and HeLa cells, the wild-type band (band a) had the expected size of 555 bp, while the mutant band (band b) was larger, indicating an aberrant splicing pattern (Fig. 1I). Sequencing results from pcDNA3.1 revealed that band a represented normal splicing, while band b retained intron12, leading to an abnormal exon-intron configuration (Fig. 1J–L). Sequencing of pcMINI-C splicing products confirmed that band a represented the normal splicing pattern, whereas band b indicated an aberrant splicing pattern involving intron A and intron12 (Figs. S1A–D). These findings provide evidence that the c.1336-2A > G variant significantly disrupts splicing, affecting the processing of *SLC34A3* mRNA. Figure 1M depicts the renal localization of SLC34A3/NaPi2c in proximal tubules.

After the genetic test, the patient stopped taking calcitriol and did not supplement with phosphate. Subsequent laboratory examinations conducted after four months revealed that her serum phosphate levels remained within the range of 0.69–0.76 mmol/L. Her urine calcium excretion had decreased to 5.91 mmol/24 h, while urine phosphate levels were measured at 12.93 mmol/L. However, after withdrawal of the calcitriol and phosphate supplementation for ten months, her serum phosphate decreases to 0.57 mmol/L. Consequently, she resumed oral phosphate supplementation to manage her phosphate levels effectively.

HHRH, a rare inherited disorder impacting the kidneys and bones, was first described in 1985.^2^ However, documented cases did not surface until 2014, with just three reported in China. HHRH results in abnormally low serum phosphate levels, leading to conditions like rickets and bone abnormalities. It is essential to differentiate HHRH from other forms of hypophosphatemia, such as X-linked hypophosphatemia, autosomal dominant hypophosphatemic rickets, autosomal recessive hypophosphatemic rickets, and tumor-induced osteomalacia, as active vitamin D is the primary treatment for most hypophosphatemia types, except HHRH. Notably, using active vitamin D in HHRH can cause kidney stone development or worsen pre-existing ones. Prior research indicated that even homozygotes will not develop kidney stones unless inappropriately given active vitamin D.^3^ Moreover, renal stones are uncommon despite high urinary calcium due to ineffective use of active vitamin D.

Genetic testing is essential for HHRH diagnosis, revealing mutations in the SLC34A3 gene, particularly affecting multiple exon site. In our patient, two mutations are present: c.575C > T p.S192L, a known mutation that replaces serine at position 192 with leucine,^4^ and a novel intronic mutation, *SLC34A3* c.1336-2A > G, impacting gene splicing, which may disrupt protein production during translation and affect mRNA stability and reliability. The pathogenic site in the proband's previous clinical genetic examination went unidentified, likely because the *SLC34A3* c.1336-2A > G mutation is located within an intron, which tends to have lower coverage in previous sequencing techniques. Additionally, pathogenic sites within introns have not been widely reported in the literature. Unfortunately, due to the misdiagnosis as X-linked hypophosphatemia and subsequent treatment with active vitamin D, the patient experienced recurring kidney stones. After receiving a correct diagnosis, discontinuing active vitamin D led to a significant reduction in the patient's urine calcium levels.

Phosphate supplementation is a promising HHRH treatment, as it significantly increases serum phosphate levels in HHRH patients, likely due to enhanced gastrointestinal phosphate absorption, partially linked to increased 1,25(OH)D3 levels, consistent with the studies of Yamamoto et al on Japanese patients.^5^ Interestingly, the patient in our case study maintained normal serum phosphate levels for almost ten months without phosphate supplementation after discontinuing active vitamin D, possibly due to a high-phosphate diet and increased intestinal 1,25(OH)D3 activity.

Our findings emphasize the crucial need for further HHRH research, with the identified mutation patterns, especially the recurring c.575C > T p.S192L mutation, offering valuable starting points for targeted treatments and improved diagnostics. Future studies should delve into understanding the precise disease mechanisms and potential therapies, as well as exploring the correlation between these mutations and clinical outcomes.

## Ethics declaration

All procedures involving human participants were conducted in accordance with the ethical standards of the institutional and national research committees and the 1964 Helsinki Declaration and its later amendments or comparable ethical standards. This study was approved by the ethics committee of Qilu Hospital of Shandong University (No. KYLL-2019-2-111). All participants signed informed consent documentation voluntarily, and the ethics committee reviewed the documentation.

## Conflict of interests

The authors declared no conflict of interests.

## Funding

This research was funded by the National Natural Science Foundation of China Youth Fund (No. 82200918) and the Natural Science Foundation of Shandong Province Youth Fund (China) (No. ZR2021QC111).
