# Supplementary material for: Newly identified intronic and known pathogenic point mutations in SLC34A3/NPT2c cause hereditary hypophosphatemic rickets with hypercalciuria
Source: Genes Dis. 2024 May 7;12(2):101318. doi: 10.1016/j.gendis.2024.101318 (PMC11614792; doi:10.1016/j.gendis.2024.101318)
Supplement: Multimedia component 1 [file mmc1.docx]

**Supplementary Data**

**Additional results**

**
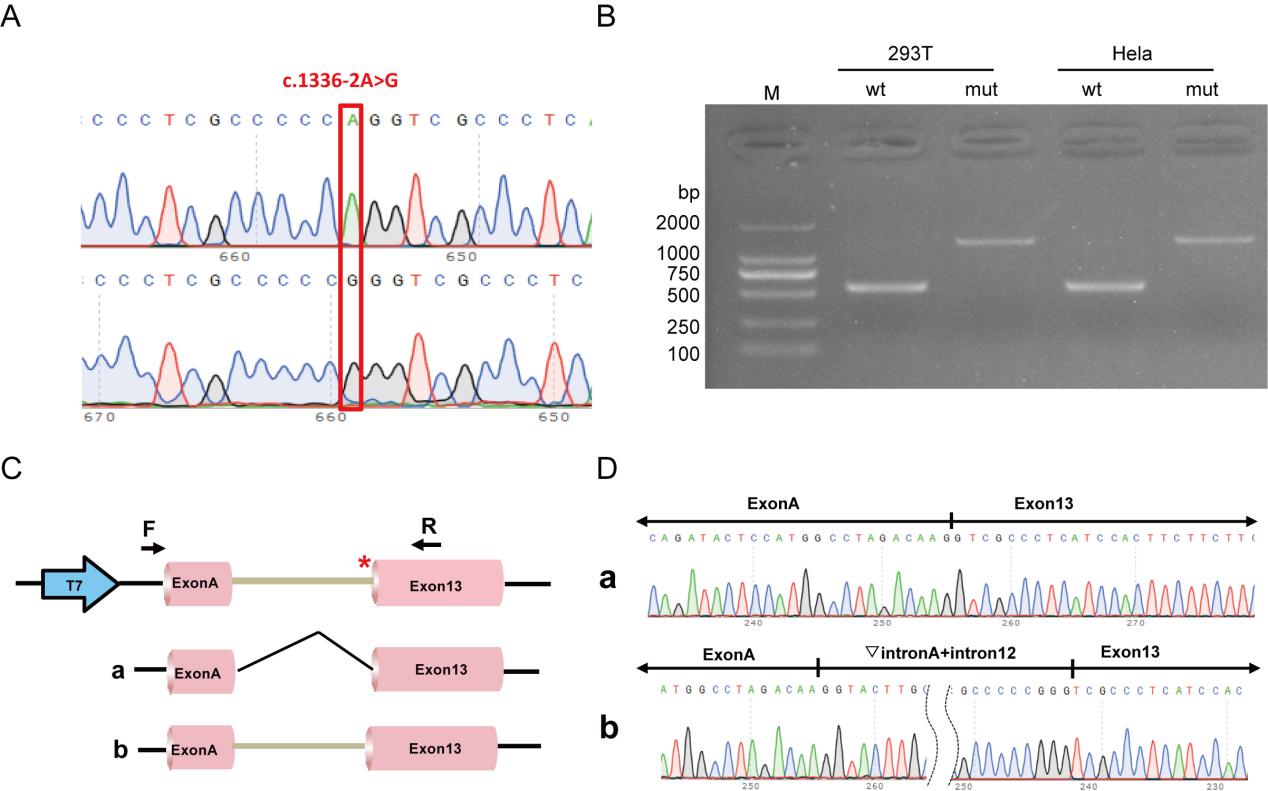
**

**Figure S1. Minigene assay results using *pcMINI-C* vectors**

1. D. Minigene assay using *pcMINI-C* vector in 293T and Hela cells.

A:Sequencing results of *pcMINI-C-SLC34A3-WT* and *pcMINI-C-SLC34A3(1336-2＞G)*. B. Agarose gel electrophoresis of the PCR products in 293T and Hela cells (band a). C. Schematic representation of the construction of *pcMINI-C-SLC34A3-WT* and *pcMINI-C-SLC34A3(1336-2＞G)* splicing modes. Modified pcDNA3.1 vectors with dual promoters (CMV promoter and T7 promoter) were used for vector construction. Both *pcMINI-C-SLC34A3-WT* and *pcMINI-C-SLC34A3(1336-2＞G)* contain exon 12, intron/exon boundaries, and exon 13. Exon 12 and exon 13 are regions with strong splicing recognition used for the splicing study. D. Sequencing results of the PCR products using designed primers.

**MATERIALS AND METHODS**

**Patients and data collection**

The proband’s medical history was evaluated, and physical and biochemical assessments were performed at the Qilu Hospital of Shandong University. Blood samples were taken after a 12-hour overnight fast. Additionally, a 24-hour urine collection was carried out to determine calcium and phosphorus concentrations. To shed light on the genetic underpinnings of the condition, a genetic analysis was conducted, coupled with intricate protein modeling techniques.

All procedures involving human participants were conducted in accordance with the ethical standards of the institutional and national research committees and the 1964 Helsinki Declaration and its later amendments or comparable ethical standards. This study was approved by the ethics committee of Qilu Hospital of Shandong University (No: KYLL-2019-2-111). All participants signed informed consent documentation voluntarily, and the ethics committee reviewed the documentation.

**Genetic testing**

Upon obtaining informed consent from the patient's family members, 2 mL of peripheral blood was meticulously drawn from the proband, her parents, and her son. The collected blood was anticoagulated using ethylenediaminetetraacetic acid (EDTA) similar to the previously study[6]. Subsequently, genomic DNA extraction was carried out using the QIAamp whole-blood DNA kit (Qiagen, Germany), following the manufacturer's recommended protocols. To unravel the genetic underpinnings of the condition, we employed whole-exome sequencing (WES) and conducted mutation analyses, in alignment with procedures detailed in our prior study. All testing and sequence analyses were conducted by Jinan AXZE Medical Test Laboratory, a collaborative partner of Qilu Hospital of Shandong University.

***In silico* analysis of SLC34A3 variants**

For the predictive modeling of three-dimensional (3D) structures, we employed the Iterative Threading ASSEmbly Refinement (I-TASSER) approach, complemented by PyMOL drawing analysis. This methodology allowed us to visualize and analyze both the wild-type *SLC34A3* (*SLC34A3-WT*) and its variant (*SLC34A3* p.Ser192Leu) at the molecular level. Through the threading method, we generated 3D models of these proteins and conducted an extensive side-by-side comparison of their final structures. To further enhance our understanding, we harnessed the Adaptive Poisson–Boltzmann Solver (APBS) within PyMOL to assess the surface charge distribution of both *SLC34A3-*WT and the variant (*SLC34A3* p.Ser192Leu). This integrated approach allowed for a comprehensive examination of structural and electrostatic differences between the two protein variants.

**Minigene splicing assay**

A minigene assay was meticulously constructed and executed in accordance with established protocols to investigate how the *SLC34A3* variants impact splicing patterns. We used *pcMINI-C* and *pcDNA3.1* vectors obtained from Bioeagle Biotech Company, Ltd., Wuhan, China. The minigene *pcMINI-C-SLC34A3-wt/mut* was constructed by inserting intron 12 (539 bp)–exon 13 (603 bp) into the pcMINI-C vector. The vector contains the general sequence ExonA-intronA-MCS.

After transfecting the cells, we analyzed ExonA-Exon13 for splicing errors. *pcDNA3.1-SLC34A3-wt/mut* was constructed by inserting exon 12 (125 bp)–intron12 (1220 bp)–exon13 (603 bp) into the pcDNA3.1 vector. Transfected cells were assessed for Exon12-Exon13 splicing errors. Mutant constructs containing the c.1336-2A>G variants were generated by PCR using primers for the mutants(Table S2). The WT and mutant plasmids were directly confirmed by Sanger sequencing and transfected into HEK293T and HeLa cells. HEK293T and HeLa cells were seeded at 3.0 × 10^5^ cells per well in a 6-well plates and transfected the next day. 3μg of the plasmid were transfected in each well using 6 μl Lipofectamine 2000 (Invitrogen, Carlsbad, CA, USA) following the manufacturer’s protocol. Twenty-four hours post-transfection, we performed total RNA extraction using an RNA extraction kit (RC112; Vazyme) and subsequently conducted reverse transcription using the Hiscript III RT SuperMix for qPCR (R323, Vazyme).The resultant cDNA was subjected to PCR amplification, and the resulting products were analyzed through 1.5% agarose gel electrophoresis. Further validation was accomplished via Sanger sequencing conducted by Bioeagle Biotech Company, Ltd. in Wuhan, China.

**Table S1. Laboratory test results of the proband and other family members**

|  | I-1 | I-2 | II-1（after treatment) | III-1 |
| --- | --- | --- | --- | --- |
| *SLC34A3* c.1336-2A>G | wt | het | het | het |
| *SLC34A3* c.575C>T | het | wt | het | wt |
| Serum Phosphate (mmol/l) | 0.93 | 0.92 | 0.69 | 1.65 |
| Serum Phosphate Normal Range (age-specific) (mmol/l) | 0.6-1.6 | 0.6-1.6 | 0.6-1.6 | 1.25-1.93 |
| Serum calcium (mmol/l) | 2.23 | 2.35 | 2.38 | 2.61 |
| Serum calcium Normal Range (age-specific) (mmol/l) | 2.11-2.52 | 2.11-2.52 | 2.11-2.52 | 2.1-2.8 |
| PTH (pg/ml) (15–65 pg/ml) | 34.64 | 46.95 | 24.35 | 27.47 |
| 25(OH)D (ng/ml) (>30 ng/ml) | 36.19 | 20.67 | 23.09 | 54.86 |
| Serum Alkaline Phosphatase (U/L) | 79 | 71 | 152 | 269 |
| Serum Alkaline Phosphatase Normal Range (age-specific) (U/L) | 45-125 | 45-125 | 45-125 | 143-406 |
| Serum Creatinine (µmol/l) (62–115 µmol/l) | 53 | 81 | 69 | 28 |
| Nephrolithiasis | No | No | Yes | No |
| Rickets | No | No | Yes | No |

**Table S2: Primer sequence for minigene splicing assay**

| Primer name | **primer sequence**（5’→3’） |
| --- | --- |
| pcDNA3.1-SLC34A3-BamHI-F | GCTCGGATCCatgGGGGTCGGGGTGATCAGTCTG |
| pcDNA3.1-SLC34A3-EcoRI-R | TGCAGAATTCCCAGATAACAGCCTGGTTTA |
| SLC34A3-mut-F | agccccctcgcccccGGGTCGCCCTCATCCA |
| SLC34A3-mut-R | TGGATGAGGGCGACCCgggggcgagggggct |
| pcMINI-C-SLC34A3-EcoRI-R | TGCAGAATTCCCAGATAACAGCCTGGTTTA |
| pcMINI-C-SLC34A3-BamHI-F | GCTCGGATCCcttgcctctgccctgtctct |
| 2965-SLC34A3-F | CGTGCACAGAGAAGAACAGC |
| 3329-SLC34A3-F | ACAGTCATCAATGCGGgtga |
| 5964-SLC34A3-R | tgatgggactccagtcccca |
| 6202-SLC34A3-R | ggtctcattctccgtggcag |
| pcDNA3.1-R(BGH-R) | TAGAAGGCACAGTCGAGG |
| pcDNA3.1-F | CTAGAGAACCCACTGCTTAC |
| SLC34A3-RT-R | CTGGTTACTGTCCTGCAGCG |
